# Moral courage, moral sensitivity and safe nursing care in nurses caring of patients with COVID‐19

**DOI:** 10.1002/nop2.903

**Published:** 2021-05-04

**Authors:** Masoud Khodaveisi, Khodayar Oshvandi, Saeid Bashirian, Salman Khazaei, Mark Gillespie, Seyedeh Zahra Masoumi, Fateme Mohammadi

**Affiliations:** ^1^ Department of Community Health Nursing School of Nursing and Midwifery Chronic Diseases (Homecare) Research Center Hamadan University of Medical Sciences Hamadan Iran; ^2^ Department of Medical Surgical Nursing School of Nursing and Midwifery Mother and Child Care Research Center Hamadan University of Medical Sciences Hamadan Iran; ^3^ Department of Public Health, Social Determinants of Health Research Center Hamedan University of Medical Sciences Hamadan Iran; ^4^ Department of Epidemiology, School of Public Health Hamadan University of Medical Sciences Hamadan Iran; ^5^ School of Health Nursing and Midwifery University of the West of Scotland Paisley Scotland; ^6^ Department of Midwifery, Mother and Child Care Research Center, School of Nursing and Midwifery Hamadan University of Medical Sciences Hamadan Iran; ^7^ Department of Nursing, School of Nursing and Midwifery, Chronic Diseases (Home Care) Research Center, Autism Spectrum Disorders Research Center Hamadan University of Medical Sciences Hamadan Iran

**Keywords:** COVID‐19, moral courage, moral sensitivity, nurses, patients, safe nursing care

## Abstract

**Aim:**

Evaluation the moral courage, moral sensitivity and safe nursing care in nurses caring of infected patients by the COVID‐19.

**Design:**

This study employed cross‐sectional research.

**Methods:**

520 nurses caring for COVID‐19 patients in 5 hospitals were selected via convenience sampling. They completed questionnaires online. Data were analysed by SPSS software version 22.

**Results:**

Findings showed that moral courage has a strong and direct correlation with moral sensitivity (*p* < .001, *r* = 0–.70) and safe nursing care (*p* < .001, *r* = 0–.74). Variables of moral sensitivity, safe nursing care, work experience, age and employment status can predict 64.76% of the variance in moral courage in these nurses. Nursing care of patients with COVID‐19 have reported high moral courage in recent months, and in spite of the numerous tensions and stresses in terms of caring these patients during this relative long period, they are still diligent in providing safe and high sensitive care to these patients.

## 1 INTRODUCTION

Nurses are one of the most important members among the caregiver teams whose beliefs and attitudes have a tremendous impact on the performance and quality of care (Shahriari et al., 2013). Caregiving is the essence of nursing; it is also the main exclusive focus on nursing performance (Blasdell, 2017; Gregg & Magilvy, 2004), so that nursing is a care profession with six principles of compassion, trust, commitment, competence, relationship and courage (Ebadi et al., 2020; Thorup et al., 2012). In the meantime, moral courage plays a significant role in providing systematic and high‐quality care (Numminen et al., 2019), due to encountering with dying and poor patients, entails invasive care, tackling with the newfound diseases and emergencies which all emerge courage a sensitivity for caregivers as the nurses (Bickhoff et al., 2016; Hawkins & Morse, 2014; Numminen et al., 2019; Sadooghiasl et al., 2018).

## 2 BACKGROUND

Moral courage is defined as courage to act according to one's own ethical values and principles even at the risk of negative consequences for the individual (Sadooghiasl et al., 2018). Moral courage helps caregivers to provide professional care to the patient, families and society in addition to having human qualities. (Hamric et al., 2015) Nurses, due to their professional nature, need moral courage to provide principled care to avoid the eventual committing immoral acts (Gallagher, 2011). Moral courage causes caregivers to adhere to the principles and values of professional ethics in some situations such as protecting the patient's privacy, giving bad news and caring for an infected patient (Gallagher, 2011; Gregg & Magilvy, 2004). But sometimes the fear of receiving unfriendly reactions from co‐workers, losing a job, violence and reduction in wages and on the contrary some benefits make them refuse to engage in moral acts and consequently experience moral distress, depression, guilt and anger, powerless and worthless feelings (Lachman et al., 2012; Shareinia et al., 2018). Therefore, to provide safe care, in addition to moral courage, health care providers need to be aware of ethical principles and have moral sensitivity (Lachman et al., 2012). Moral sensitivity definitions as the nurses' knowledge of patients' vulnerability and predict the results of moral decision‐making in patients, it enables them to make a moral decision for patients (Amiri et al., 2020). Therefore, moral sensitivity is one of the main pillars of nursing professional competence that leads to the development of patience, calmness and caregivers responsibility (Bijani et al., 2020). Studies show that caregivers with moral courage and moral sensitivity commitment provide safe care for patients and they are sensitive to the physical and psychological needs of patients and actively identify the needs and problems of patients and find a way to solve these problems and provide safe care (Mohammadi et al., 2020). Therefore, there is a close relationship between moral courage with moral sensitivity and safe care, that, especially in the new crisis of COVID‐19, can affect the performance of nurses caring for patients with COVID‐19. In the meantime, the new emerging disease COVID‐19 started in China and then, it became pandemic in all countries and caused over 8.3 million people in the world to get infected by this disease in the past 3 months, over 224,000 of whom have lost their life following this issue (Coronavirus, 2019; Lai et al., 2020). Iran outbreak of COVID‐19 is complex, at the beginning of the pandemics Iran was the third country with the highest number of reported COVID‐19 cases after China and Italy, whereas it has been heavily hit by the virus and now facing the third wave of the COVID‐19 outbreak compared with other countries that are currently facing the fourth wave. Although the majority of Iranians wear masks, inappropriate economic situation causes very few restrictions on work, activity and travel all around the cities of Iran; therefore, large number of people use public transportation every day, and the streets are crowded. In addition, all employees spend their time at work in person every day as the telecommunication is rare to occur (Nemati et al., 2020). These factors have been the remarkable causes of infection so that about 530,500 people in Iran have been infected with COVID‐19 and over 30,500 of them have lost their lives (Education, 2020; Nemati et al., 2020). World Health Organization (WHO), in January 2020, introduced COVID‐19 pandemic as a Public Health Emergency of International Concern (PHEIC) (Liu et al., 2020). According to reports from the same organization, nurses are the first health advocates against the crisis caused by the COVID‐19, who experience severe stress and psychological distress. As an inclusive group in the health care system, nurses play a significant role for the evolution and care development, treatment, improvement and health promotion in these patients. In addition, dealing with critically poor and dying patients with COVID‐19, who have high contagious ability since there is no definitive treatment, exposes the nurses with numerous ethical tensions and challenges to provide safe care (Numminen et al., 2019). In regard with this difficulty, studies have examined the stress and anxiety of nurses taking care of the mentioned patients with COVID‐19. Accordingly, Stuijfzand et al., (2020) state that healthcare professionals during epidemic and pandemic (Severe Acute Respiratory Syndrome(SARS), Middle East respiratory syndrome coronavirus (MERS), Ebola Virus Disease) experience a great deal of mental health problems including psychological distress, insomnia, alcohol/drug misuse and symptoms of post‐traumatic stress disorder (PTSD), depression, anxiety, anger and burnout (Stuijfzand et al., 2020). Also, Lehmann et al. (2015) represent that health care professionals reported Depression, stress, social isolation and job fatigue during Ebola outbreak. Social isolation has been largely reported due to parental stress and concerns related to the disease transmission amongst the children and other family members. On the other hand, although job stress and fatigue can affect nurse`s tolerance, they have overcome these work pressures and tensions with team working and observing the principles of infection control (Lehmann et al., 2015). A study which has already been conducted by Liu et al., (2020) confirms that compared to the other medical staff members nurses caring COVID‐19 patients experience higher levels of anxiety and depression because not only should they struggle with various difficulties in the performance of their professional duties, but as a member of their families they also should shoulder their daily responsibilities (Liu et al., 2020). Similarly, the study of Roy (2020) showed that nurses are experiencing high degrees of stress, anxiety, depression and post‐traumatic stress disorder (PTSD) in the COVID‐19 crisis. Therefore, it seems to be necessary to conduct a study to investigate the relationship between moral courage, moral sensitivity and safe care in nurses of patients with COVID‐19 in Iranian society.

## 3 METHODS

### 3.1 Study design and setting

This study is a cross‐sectional research. The conducted investigation is based on the strengthening the reporting of observational studies in epidemiology statement (STROBE), that is checklist for observational research (Appendix S1), from March to June 2020. The two following aims were examined in study “evaluation of moral courage, moral sensitivity and safe care in nurses who take care of COVID‐19 patients” and “investigating the relationship between moral courage, moral sensitivity, safe care and demographic characteristics in nurses who take care of COVID‐19 patients.”

### 3.2 Participants and sampling

Sample size has been estimated 520 samples in this study according to the past study of Labrague, et al. With *β* = 80% and *α* = 0.05 and taking into account the 10% drop in each group (Labrague & De los Santos, 2020). The participants were selected via convenience sampling. Ultimately Nurses who worked in the infectious ward and provided care to COVID‐19 patients in 5 hospitals affiliated with University of Medical Sciences in the west of Iran. They were invited and selected via convenience sampling to participate in the study. The inclusion criteria were: being willing to participate, being one of the members of the hospitals where have been devoted to COVID‐19 patients, and having at least 1 month of work experience in the particular wards for patients with COVID‐19. The participants who failed to answer more than half of the items on their questionnaires or did not return their questionnaires were excluded. The participants were asked to complete and submit the questionnaires – a personal (demographic) characteristics questionnaire, an occupational burnout scale, resilience scale, and, if they were parents, the parenting stress scale – online. The researchers sent emails and reminder messages to the participants, so that the majority of the questionnaires (90%) were completely gathered in June. 420 of the subjects completed and returned the questionnaires via e‐mail or a social network. Thus, the response rate was 77.88%, the nurses' reasons for not being participated in this study were high prevalence of COVID‐19 in Iran, heavy workload and intensive shifts, also infected with COVID‐19.

### 3.3 Questionnaire

#### 3.3.1 Demographic information questionnaire

The questionnaire included age, gender, marital status, employment status and work experience, the average number of monthly shifts, shift work, financial condition and education.

#### 3.3.2 Nurses' moral courage questionnaire

This questionnaire was designed and validated by Sadoughi et al. in 2015. This 20‐item questionnaire consists of three dimensions of moral self‐fulfilment (9 questions), risk‐taking (8 questions), and the ability to defend the right (3 questions), which is graded according to the five‐point Likert scale from always (score 1) to never (score 5). The score of each item is obtained by multiplying the Likert score by the value of the item. The questionnaire has a minimum score of 102 and a maximum score of 510. In this questionnaire, low moral courage was considered 102–238, medium moral courage 239–374 and high moral courage 375–510. Content validity obtained by determining the content validity index is (CVI = 0.87), its internal consistency by calculating Cronbach's alpha coefficient is 0.88 and its consistency equals 0.87 by doing the test–re‐test and calculating the intra‐class correlation coefficient (Sadooghiasl, 2016).

#### 3.3.3 Moral sensitivity questionnaire

This questionnaire was designed by Lutzen et al. containing 28 questions in 6 subscales: (1. level of respect for the patient's independence, 2. levels of knowledge about how to communicate with the patient, 3. levels of professional competence, 4. experiences of moral issues and conflicts, 5. application of ethical concepts in moral decisions and 6. honesty and benevolence) based on 7‐point Likert scale (1 strongly disagree to 7 agree). The design of the Likert scale is suitable for 7 degrees (1 strongly disagree to 7 strongly agree). This questionnaire is scored in three spectrums of moral sensitivity: low (28–84), medium (85–141), and high (142–198). This questionnaire has content and face validity in Iran and reliability with a Cronbach's alpha coefficient of 0.83 (Hassanpoor et al., 2011).

#### 3.3.4 Evaluating safe nursing care questionnaire

This questionnaire was designed by Rashvand et al. to evaluate safe nursing care, based on the context of the Iranian care system. This questionnaire has 32 questions in 4 sections. The first part is related to the Evaluation of nursing Skills (16 questions), the second part is Assessing the patient's psychological needs (4 questions), the third part is the Assessing the patient's physical needs (7 questions), and the fourth part is the assessment of the nurse's teamwork (5 questions); Answers to all questions are scored on a 5‐point Likert from never (1 point) to forever (5 points). The weight of questions 14, 18, 19, 20 and 32 is equal to 1; the weight of questions 2, 3, 4, 5, 7, 10, 11, 12, 13, 15, 16, 17, 21, 26 and 30 is 2. The weight of questions 1, 6, 8, 9, 23, 24, 25, 27, 29 and 31 is 3, and the weight of questions 28 and 22 is 4; therefore, the obtained number was multiplied by the weight of the question, and the final number was used for the analysis. A score of 1 to 4 indicates poor performance; a score of 2 to 4 indicates moderate performance, and a score of 1 to 4 shows good performance. The calculated reliability of this tool using Alpha Cronbach's method was 0.97 that indicates its optimal reliability (Rashvand et al., 2017).

### 3.4 Statistical methods

In this study, the collected data will be analysed with SPSS software version 22. For this purpose, descriptive statistics (frequency, percentage, mean and standard deviation) were used. Chi‐square and independent *t* test were also used to investigate the relationship between moral courage and the level of moral sensitivity and safe care and demographic information. The significance level was considered *p* < .05. Then the demographic variables and moral sensitivity and safe care associated with moral courage (*p* < .25) were entered into the multiple linear regression model with a backward strategy. The researcher evaluated before performing multiple linear regression, hypotheses including normality of data, homogeneity of variance and independence of the residual.

### 3.5 Ethics approval and consent to participate

The study design was approved by the Ethics Committee of the Hamadan University of Medical Sciences (Umsha.rec.1399.524). Also at the beginning of study the researcher introduced herself and explained the goals of the study and assured that all information would remain confidential and that they could withdraw from the study at any time. Finally, the written informed consent was obtained from all the participants after providing them with sufficient information on the study.

## 4 RESULTS

### 4.1 Demographic information

Participants in this study were in the age ranging from 25 to 56 years with a mean age of 39.04 ± 2.13. Also, most of the nurses participating in this study (68.14%) 276 had a bachelor's degree in nursing, contractual employment 205 (50.61%), had 10 years of experience with an average of 34 shifts per month. Findings also showed that there is a statistically significant relationship between moral courage and work experience, age and employment status (Table 1).

**TABLE 1:** The participants' demographic characteristics and moral courage score

Demographic variables	*N* (%)	Moral courage Mean ± *SD*	*p* Value
Age (year)			
23–33	189 (46.66)	376.76 ± 1	.0219
34–44	169 (41.73)	398.08 ± 1	
45–55	47 (11.61)	312.98 ± 2	
Gender			
Female	220 (54.32)	378.63 ± 2	.432
Male	185 (45.68)	382.53 ± 2	
Marital status			
Single	169 (41.73)	383.76 ± 2	.361
Married	236 (58.27)	343.54 ± 1	
Education			
Diploma of nursing	44 (10.88)	378.24 ± 1	.453
Bachelor of nursing	276 (68.14)	389.12 ± 1	
Master of nursing	85 (20.98)	388.15 ± 1	
Employment status			
Employment	208 (49.54)	393.98 ± 1	.0381
Contract employment	212 (50.47)	387.87 ± 1	
Work experience			
>10 years	150 (35.72)	396.02 ± 2	.017
10–20 years	197 (46.90)	432.14 ± 2	
20–30 years	73 (17.38)	375.23 ± 2	

### 4.2 Moral courage, moral sensitivity and safe nursing care in nurses

Nurses participating in this study reported a moral courage score of 473.33 ± 1.64, a moral sensitivity score of 178.61 ± 98 1.98 and a safe nursing care score of 298.53 ± 2.27 in caring of patients with COVID‐19 (Table 2).

**TABLE 2:** The means and standard deviations of the participants' Moral courage, Moral sensitivity and Safe nursing care

Variable	dimensions	Mean ± SD (Each dimension)	Mean ± SD (Total)
Moral courage	Moral self‐actualization	409.31 ± 1	473.33 ± 1.64
	Risk taking	396.66 ± 1	
	Ability to defend the right	381.07 ± 1	
Moral sensitivity	Respect rate for the service user's independence	144.11 ± 2	178.61 ± 1.98
	Awareness of how to communicate with the patient	156.83 ± 1	
	The amount of professional knowledge	187.74 ± 1	
	Experiencing moral problems and conflicts	163.09 ± 1	
	Applying moral principles in ethical decisions	153.92 ± 1	
	Honesty and benevolence	174.28 ± 1	
Safe nursing care	Evaluation of nursing Skills	356.65 ± 2	298.53 ± 2.27
	Assessing the patient's psychological needs	297.87 ± 1	
	Assessing the patient's physical needs	309.54 ± 2	
	Assessing nurses' teamwork	341.15 ± 2	

### 4.3 The relationship between moral courage, moral sensitivity and safe nursing care in nurses

Findings in this study revealed that there is a strong and direct correlation between moral courage with moral sensitivity (*r* = .91, *p* < .001) and moral courage with safe nursing care (*r* = .89, *p* < .001) in nurses caring of patients with COVID‐19. Also, the reports showed a close correlation between the score of moral sensitivity and safe nursing care (*r* = .84, *p* < .001) (Table 3).

**TABLE 3:** Relationship between moral courage, moral sensitivity and safe nursing care in nurses

Moral courage	Moral sensitivity	*r* = .91	*p* < .001
Moral courage	Safe nursing care	*r* = .89	*p* < .001
Moral sensitivity	Safe nursing care	*r* = .84	*p* < .001

### 4.4 Predictors of moral courage in nurses caring for patients with COVID‐19

The variable of moral sensitivity, safe nursing care, work experience, age and marital status with which had a p‐value of smaller than 0.25 were entered into multiple linear regressions with the backward technique. These variables remained in the model and accounted for about 64.76% of the moral courage variance in the nurses who provided care to COVID‐19 patients (Table 4).

**TABLE 4:** The predictor variables of moral courage in nurses caring for patients with coronary heart disease

Factors	Non‐standard coefficients		Standard coefficients	*T*	*p* Value
	B	Standard deviation	*β*		
Moral sensitivity	−0.687	2.78	−0.701	−3.12	.001
Safe nursing care	0.646	2.67	0.658	2.78	.001
Job experience	0.387	2.43	0.453	2.96	.021
Age	0.298	2.23	0.378	2.54	.026
Employment	0.182	2.12	0.232	2.21	.035
Adjusted *R* ^2^: 64.76%					

## 5 DISCUSSION

This study showed nurses have reported high levels of moral courage, sensitivity and safe nursing care. Although several studies have examined the work stress, knowledge and awareness of nurses caring for patients with COVID‐19, no article has been found to investigate moral courage, moral sensitivity and safe nursing care in the 2019 COVID Crisis. Also, the researcher had little access to writing a comprehensive discussion; thus, the researcher used other articles that measured the level of moral courage, moral sensitivity and safe nursing care for specific patients.

The reported score of nurses' moral courage in this study was 473.33 ± 1.64, which indicates the high moral courage of nurses taking care of patients with COVID‐19. Consistent with the findings of this study, other studies have shown that nurses, especially in infectious wards and intensive care units, have reported high moral courage (Moosavi et al., 2017; Moosavi & Izadi, 2017; Mahdaviseresht et al., 2015). While Hanna et al. rated the moral courage of nurses as moderate (Hannah et al., 2011), Day et al. estimated weak moral courage in nurses. This difference can be due to different cultural and organizational contexts, observance of administrative hierarchy, legal consequences such as compensation to patients, and personal characteristics of nurses such as motivation, attitude towards care, religious beliefs and values, etcetera (Corley, 2002; Kidder, 2005; Mahdaviseresht et al., 2015; Shoorideh et al., 2015). On the other hand, the score of moral courage of nurses responsible for COVID patients was higher than other related studies in this regard; The probable reason for this difference could be the sudden occurrence of COVID‐19 disease with high transmission power and nurses' sense of responsibility for a useful and constructive presence to save the patient's lives and keeping humanity in this global crisis.

Moral sensitivity in this study was estimated to be 178.61 98 1.98 and at a high level, while the findings of Amiri et al.'s study that was in the same line with the present study have estimated high moral sensitivity for nurses caring for patients in internal wards as well (Amiri et al., 2019). Consistent with the findings of this study, Ohnishi et al. also stated that the moral sensitivity of nurses working in the psychiatric ward in Japan and Finland is high (Ohnishi et al., 2019). But other studies have also reported that the moral sensitivity of nurses in caring for patients is moderate or even weak (Borhani et al., 2013; Izadi et al., 2013). Possible causes of this discrepancy can be cultural and organizational differences and, above all, the differences in the type of disease and care departments. That is due to the type and physical and mental condition of patients that can affect moral sensitivity in nurses in a way that nurses caring for patients in intensive and psychiatric wards have reported more moral sensitivity than the nurses in other units (Ohnishi et al., 2019; Mahdaviseresht et al., 2015). On the other hand, the sudden occurrence of COVID‐19 and its high contagious power caused some nurses to be reluctant to continue working in hospitals and even leave hospitals, but nurses who are still in the hospitals and have taken care of these patients, probably more carefully and precisely and considering all the characteristics and conditions of these patients, have tended to take care of them by observing professional principles; consequently, they have a higher moral sensitivity in care.

The safe care score of the nurse participating in this study was 298.53 ± 2.27; therefore, reporting a reasoned level. However, some studies have investigated safe nursing care in nurses working in intensive care units and general wards (Amiri et al., 2019; Arshadi Bostanabad & Jebreili, 2015; Sa & Sah, 2019; Valentin et al., 2006). According to Nouhi et al. there is a statistically significant relationship between the type of ward and nursing performance in dimension of physical safety and clinical skills of nurses. Nurses working in intensive care units have more clinical skills than the ones working in general wards and when providing care, they observe the principles of safety care, especially in dimension of physical safety (Nouhi et al., 2016). In the line with this study, other studies show that nurses in different wards, especially intensive care units for adults and infants, pay more attention to the principles of safe care throughout the work (Baykara et al., 2015; Lützén et al., 2003; Mathur & VanderWeele, 2020). This similarity is probably because patients with COVID‐19 are usually hospitalized to intensive care units in the hospital with symptoms of respiratory distress and heart problems. On the other hand, the unknown nature of this disease has caused nurses to be more careful when caring and to follow the principles of safe care more.

The findings of this study also indicate that work experience and age are directly related to the level of moral courage of nurses participating in this study. Other studies in line with the findings of this study showed that increasing age and work experience augment the moral courage in nurses, and then they show more courage in performing care (Navidian et al., 2014; Parker, 2014). This relationship is probably due to the promotion of individual's awareness about organizational conditions, gaining professional and practical competence, and learning courageous behaviours from other colleagues (Ebadi et al., 2020; Murray, 2010; Numminen et al., 2019). On the other hand, the moral courage of nurses had a statistically significant relationship with their employment status. Nurses with contractual employment reported less moral courage, while nurses with civil employment conditions indicated higher levels of moral courage. The instability of employment conditions and receiving less salary are probably the reasons for this statistically significant difference. Because the employment condition shows the individual's relationship with the organization and, as a result, the mutual dependence of the people to the organization; therefore, in long work relationships, the individuals feel safer and more secure occupationally. Individuals who are supported organizationally exhibit more courageous behaviours, while job insecurity constitutes a barrier to courage performances (Abadi et al., 2020).

The results of the present study show a strong and direct correlation between the participants' moral courage with moral sensitivity and safe care nursing. In addition the variable of sensitivity, safe care nursing, work experience, age and marital status descripted about 64.76% of the moral courage variance in the nurses who provided care to COVID‐19 patients. Study did not find any evaluated and predicted relationship between moral courage with moral sensitivity, safe care nursing and demographic characteristic in nurses at the same time. Therefore, other studies were used that examined the relationship between moral courage with moral sensitivity and moral courage with safe care nursing or occupational burnout with demographic factors. In this regard, Mahdaviseresht et al. stated that there is an direct relationship between moral courage with moral sensitivity (Mahdaviseresht et al., 2015). Also, Chua et al. expressed moral courage was directly related to moral sensitivity (Escolar‐Chua, 2018), these findings are consistent with the results of present study. Abadi et al. stated, there was no significant relationship between the moral courage and the quality of work life and safe care (Abadi et al., 2020). The probable cause of this difference can be considered in the type of the disease that patients had been infected. In the above study, the patients did not have the disease which can be transferred to the caregivers, while the nurses in the present study looked after the patients with COVID‐19, which can transmit quickly to the caregivers, and having the moral courage is very important for caring of these patients. In addition Ebadi et al. stated nurses who worked in the intensive care units reported more moral courage than the nurses in other wards and there was correlation significant between moral courage with age, employee, shift working, position and experience (Abadi et al., 2020). Also findings in Khajevandi et al. study show there was direct correlation between moral courage with employee status (Khajevandi et al., 2020), which are consistent with the present study.

Finally, it can be stated that nurses who care for COVID‐19 patients during recent months, despite the extremely high workload with a lot of stress and tension, have reported high moral courage and sensitivity, which is the best reason to provide safe care to these patients. Therefore, considering the continuation of the COVID‐19 crisis in the coming months in Iran and the world, it is necessary that health administrators take effective measures to promote nurses' moral courage and subsequently enhance safe care nursing form for patients with COVID‐19.

### 5.1 Limitations

One of the major limitations of the present study was the relative low return rate of the questionnaires via e‐mail which could have been due to the hectic work schedules of nurses in the crisis. Moreover, the variables addressed in the present study were measured over a 6‐month period – it is suggested that future studies assess moral courage, moral sensitivity and safe nursing care in nurses who care for COVID‐19 patients in the coming months and years in other societies and larger samples in order to acquire a more accurate understanding of nurses' moral courage in this crisis. Health administrators and policy makers can use these findings to develop more comprehensive plans for the current and future crises.

## 6 CONCLUSION

In the present study, the nurses who care for COVID‐19 patients reported high levels of moral courage, sensitivity and safe care nursing. Also, the results showed that nurses' moral courage correlates with their sensitivity, safe nursing care, work experience, age and marital status: these variables predicted 64.76% of the subjects' moral courage variance. The nurse managers and policy makers of health organizations should examine and use the findings of this study in order to provide appropriate environments for nurses, also to develop more comprehensive plans for support of nurses for the current and future crises.

## 7 RELEVANCE TO CLINICAL PRACTICE

Moral courage promotes the moral sensitivity of nurses in clinical practice, and consequently, nurses who have the courage and moral sensitivity, take care of patients, even patients with infectious diseases and without definitive treatment, better and safer. Therefore, to increase moral sensitivity and moral courage and improve safe nursing care, nursing managers should pay attention to the following points in clinical practice. First, it is important to educate nurses about codes and moral sensitivity. Second supporting them to develop moral courage in nurses, so that, they can provide safe nursing care.

## CONFLICT OF INTEREST

No conflict of interest is declared by the authors.

## AUTHOR CONTRIBUTIONS

FM, SB, KO, and SZM involved in the conception of the study and designed the study. They are responsible for data collection. Then, FM, KO, SZM and SK analysed data. FM, FM, SB, M Kh and KO drafted the primary manuscript, revised and approved the final manuscript.

## ETHICAL APPROVAL

The institutional review board of the Hamadan University of Medical Science located in the west of Iran provided ethics approval (approval number: 1399.524). Also at the beginning of each interview, the researcher introduced herself and explained the goals of the study and assured that all information would remain confidential and that they could withdraw from the study at any time. The researchers provided the opportunity for participants to inform the researcher about their withdrawal from the study at any stage of the research and assured. Finally, the written consent was obtained from study participants.

## Supporting information

App S1

## Data Availability

The written consent was obtained from study participants for publishing data.
